# Information Routing Driven by Background Chatter in a Signaling Network

**DOI:** 10.1371/journal.pcbi.1002297

**Published:** 2011-12-08

**Authors:** Núria Domedel-Puig, Pau Rué, Antonio J. Pons, Jordi García-Ojalvo

**Affiliations:** Departament de Física i Enginyeria Nuclear, Universitat Politècnica de Catalunya, Terrassa, Barcelona, Spain; University of Virginia, United States of America

## Abstract

Living systems are capable of processing multiple sources of information simultaneously. This is true even at the cellular level, where not only coexisting signals stimulate the cell, but also the presence of fluctuating conditions is significant. When information is received by a cell signaling network via one specific input, the existence of other stimuli can provide a background activity –or chatter– that may affect signal transmission through the network and, therefore, the response of the cell. Here we study the modulation of information processing by chatter in the signaling network of a human cell, specifically, in a Boolean model of the signal transduction network of a fibroblast. We observe that the level of external chatter shapes the response of the system to information carrying signals in a nontrivial manner, modulates the activity levels of the network outputs, and effectively determines the paths of information flow. Our results show that the interactions and node dynamics, far from being random, confer versatility to the signaling network and allow transitions between different information-processing scenarios.

## Author Summary

Far from being silent and static, the habitat of a cell is usually composed by multiple and simultaneous signals. We can consider nutrients, hormones, temperature, light, and other stimuli as elements building a default environment in which cells grow, divide and die. This environment, which has an intrinsically fluctuating nature, is the setting in which cells process all incoming stimuli. Here we examine the role that this background activity –or signaling chatter– plays in the transmission of information in a typical human cell. We address this question using a cellular model of signal transduction that we simulate using both random and periodic stimuli. We find that the level of background chatter determines the response of the whole signaling network to external stimuli. Different areas of the network are activated by specific levels of background activity, routing the information through chatter-dependent paths. In this way, different levels of chatter allow the network to select between different responses, given the same stimulus. These features depend on the architecture and functional connectivity of a truly biological network, since we find that randomized versions of the model are incapable of showing this behavior.

## Introduction

Signal transduction, the process through which information about the extracellular environment is conveyed to the cell's interior, is a property of all living organisms. Signaling molecules stimulate their receptors, which transmit the signal downstream through a series of protein-protein interactions that ultimately modify DNA expression and protein levels [1], [2]. In this manner, external information affects cell behavior. This description of signal transduction has traditionally involved independent signaling cascades –or pathways–, in which information is linearly transmitted from membrane to nucleus. Correspondingly, experimental studies have usually analyzed pathway stimulation by single inputs, such as variations in a chemical signal (e.g. a nutrient or hormone) or a physical property (e.g. illumination or mechanical pressure). However, extracellular media often contain a complex mix of molecules that have the potential to feed the signaling network with multiple inputs simultaneously [3]. In addition, it is now known that proteins in one signaling cascade often interact with proteins of other pathways, forming a dense web of connections 4–6. Thus cells must be able to perform such complex information-processing tasks as signal integration [7]–[9] and multiplexing [10] while dealing with cross-talk [11], [12].

**Figure 1:**
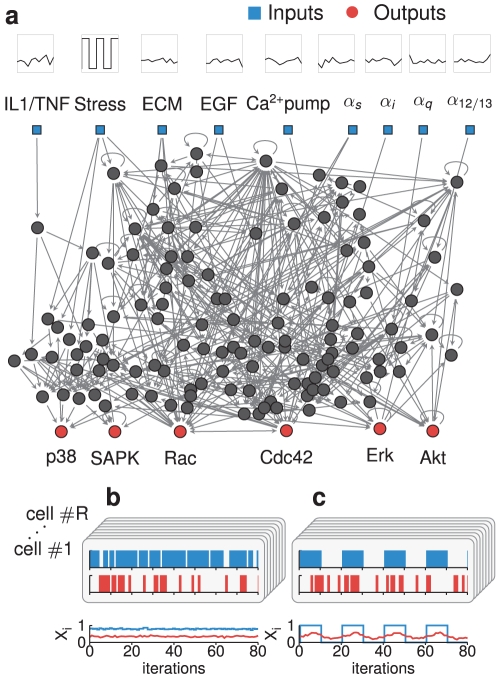
Boolean network model and simulations. (a) The Boolean network model under investigation. The network has 9 input nodes (blue squares at the top row), which we force with periodic or random signals (compare stress to the other inputs, respectively), as pictured schematically above each node, depending on the simulation performed. The network has 130 internal nodes, six of which are considered output nodes (red circles at the bottom row, while the rest of the internal nodes are represented by black circles). (b) Response of the network to a constant chatter level. Here we show a realization of the network dynamics (cell #1) in which the states of all input nodes randomly switch between 0 and 1 at a chatter level of 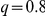. For clarity, we show the evolution of only one input node (stress, in blue) and one output node (p38, in red). The population average signal for the two nodes, calculated averaging data from R = 201 realizations, is shown at the bottom. (c) Response of the network to periodic stimulation of one input node. We show one network realization in which input node stress oscillates (period 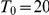), and the other input nodes are set to chatter level 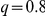.

Adding to these information-processing requirements, the signaling machinery has to cope with the fact that a cell's environment is not stationary, but subject to fluctuations [13], [14]. Here we explore the impact of these environmental fluctuations on the information processing capabilities of the signaling network as a whole. In particular, we study how transmission of information from one single input node is affected by the fluctuating background activity, or *chatter*, provided by other network inputs. To address this issue in a way that explicitly accounts for the complexity of the system under consideration, we use one of the most comprehensive dynamic models of cell signaling currently available in the literature, a recently published Boolean network for the human fibroblast that involves over 130 protein species [15] (see Figure 1a; a fully annotated version can be found in Figure S1). The dynamics of this network are implemented as a set of logic rules, an approach that, despite its simplicity, represents a good choice when building a detailed kinetic model is unfeasible. Indeed, Boolean networks have successfully been applied to modeling numerous biological processes, including gene regulation [16]–[19], cellular differentiation [20], [21], developmental patterning [22], and signal transduction [23]–[26], evidencing that sequences of cellular events can be reproduced by this type of discrete dynamic models.

Given the ubiquity of periodic oscillations in cellular processes [27]–[29], we assume that the input signal is either periodic in time (structured signal) or erratic (unstructured). By performing extensive numerical simulations, we characterize the response of the fibroblast's signaling network to such periodic input signals under different chatter levels. Our findings suggest that the level of background activity shapes the response of the entire network to the input signal, thus providing a mechanism for *context-dependent* signaling [11] in dynamic situations.

## Model

The Boolean Network (BN) model used in this work was built by Helikar and coworkers [15] to describe the signaling pathways in a prototypical human fibroblast (see Supporting Text S1 for additional details). The network, which was created by careful inspection of a large body of experimental literature, contains 9 input nodes and 130 internal nodes (see Figure 1a). The input nodes represent signals of varying nature, namely stress signals, a growth factor, a calcium channel, signaling by extracellular matrix components and by ligands that use G-protein coupled receptors. Following the original work, we consider six of the 130 internal nodes to be outputs of the network, even though they also signal to other nodes. The choice of these six species (the proteins Akt, Erk, Rac, Cdc42, SAPK and p38) as network outputs was motivated by their role in regulating well-defined cellular processes: programmed cell death (apoptosis) in the case of Akt, gene transcription for Erk, cytoskeletal regulation for Rac and Cdc42, and, finally, cellular stress for SAPK and p38.

Mathematically, the BN used here (termed *original* network hereafter) consists of 139 elements, or nodes, connected by 542 links. Nodes represent chemical species, which are assumed to be either active or inactive, and edges represent their interactions. The states of all species are updated synchronously at each iteration according to a set of node-specific deterministic rules. Hence, the state 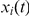 of node 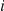 (equal to 0 if the node is inactive and 1 if it is active) is completely determined by the states of its 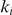 inputs at iteration 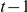 and by its logic rule 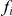 (see full details in the Supporting Text S1). Therefore, for given inputs and initial conditions of the network, the states of all nodes evolve in a deterministic and reproducible manner. To introduce the unpredictable evolution of the cell environment into the model, here we allow random fluctuations in the activity of the input nodes. We define a probability 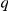 for an input to be active, and at each iteration of the network dynamics we draw the state of this input node from a Bernoulli distribution with probability of success equal to 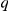 (i.e. 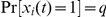 for input node 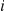). The parameter 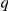 therefore determines the average level of background *chatter* in the network, which is lowest for 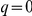 and highest for 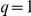. Note that 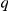 additionally controls the degree of variability in the input sequence, which is maximal at 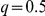, and disappears for both 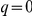 and 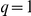.

In this context, a single realization of the network dynamics may be assumed to correspond to the behavior of a single cell, and the ensemble average of the activity over 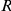 cell realizations at a fixed 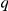 level can be regarded as the average cell population activity, 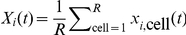. Note that 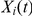 is a continuous variable representing the proportion of cells in the population with species 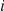 active at iteration 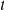. This ensemble representation, together with the chatter model introduced above, allows for a realistic description of stochastic fluctuations, as it dilutes the effect of flipping input-node states at the macroscopic level [30], provided the number 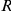 of cell realizations is large enough. In this representation, the chatter levels correspond to the population average activities of the input nodes.

The random sequences of activity states obtained for inputs with a constant chatter level provide the network with an unstructured signal that lacks temporal information. Figure 1b shows the behavior of the output node p38 (red) for a given realization of the unstructured sequences for all inputs set at chatter level 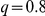 (for clarity, only the input stress is shown, in blue). All simulations below have been made for a duration of 1600 iterations, well beyond the span of any transient behavior [26] (which has been eliminated by removing the first 160 iterations before data analysis). Notice that both nodes show constant activity at the population level (see bottom plot in Figure 1b). In this case, averages over cell realizations are effectively equivalent to temporal averages. In this paper we consider an additional type of input sequence that does introduce temporal information: oscillating (structured) inputs whose states turn on and off periodically. We illustrate this case in Figure 1c, in which the input stress oscillates and the rest of inputs fluctuate with a fixed level of chatter, 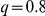 (only the input stress is shown). In this case, the average population activity of the output node p38 also oscillates at the frequency of the input, therefore recovering the temporal information supplied by the external stimulation. In the following we describe the conditions in which these structured and unstructured signals are transmitted, and the role played by background chatter during this process.

## Results

### The logic of the original network allows sensing the background chatter level

**Figure 2:**
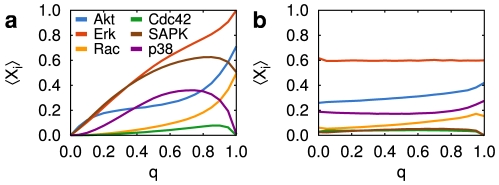
Temporal average of the population activity of the output nodes for increasing chatter levels. In these simulations, the activity of all input nodes is unstructured (i.e. the probability of the input nodes being active is set to a constant value 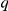). (a) Original network: the average activity of some output nodes increases monotonically, while it peaks at intermediate chatter levels for other outputs. (b) Randomized altered-logic network: the network activity is basically insensitive to chatter levels. In these randomized networks, the logic rules of the nodes are changed randomly (see Supporting Text S1 for details). The population activity was estimated from 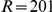 cell realizations for each network. After dismissing a transitory period of 160 iterations [26], the temporal average of the population was calculated.

To study the contribution of chatter to the network dynamics, we first consider the response of the network to *unstructured* inputs. These have been implemented with a constant chatter level 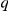 for all the input nodes. Under these conditions, the population activities of the output nodes fluctuate around a constant value that depends on the chatter level (see bottom plot in Figure 1b). In Figure 2a we show the temporal average of the population signal for all the output nodes, for increasing levels of chatter. The average population activity increases monotonously for three of the outputs (Akt, Erk and Rac) as the chatter level increases. In particular, we observe that the average activity of Erk is approximately proportional to the chatter level. On the other hand, the average population activities of the other three outputs (Cdc42, SAPK and p38), depends non-monotonically on the chatter, becoming maximal for an intermediate value of 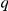. Thus, the original network responds to background chatter in a nontrivial manner.

We now ask to what extent the effects of constant chatter described above can be attributed to the specific connectivity architecture of the fibroblast network being used here. In order to address this issue, we generate a family of random networks that maintain the topology (i.e. the setting of nodes and links) of the original network, while permuting randomly the update rules, thus changing the logic of each node (see Figures S2 and S3, and accompanying Supporting Text S1 for the full details). The results obtained from multiple realizations of this randomized *altered-logic (AL)* version of the network show that its response is, in general, not sensitive to the chatter level (Figure 2b). Therefore, the responsiveness to chatter is not guaranteed solely by the network topology, but seems to require a particular type of logic rules governing the dynamics of the nodes. To check whether this is indeed the case, we generate a second family of randomized networks, keeping now unaltered both the topology of the original network *and* the logic rules of the nodes, but randomly reassigning the inputs of each of the update rules (see Figure S2). This randomization is less severe than the previous one, since it maintains the type of logic rules in the network. We observe that networks of this *altered-input (AI)* family are sensitive to chatter levels in a similar way to the original experimentally-based network (Figures S4 and S5). Taken together, these results reveal that the biologically realistic network studied here responds in a nontrivial manner to a constant level of background chatter, and it is the distribution of logic rules of the network nodes, which is far from random (see network properties in the Supporting Text S1), that determines this responsiveness.

### Background chatter enhances the network response to periodic stimulation

**Figure 3:**
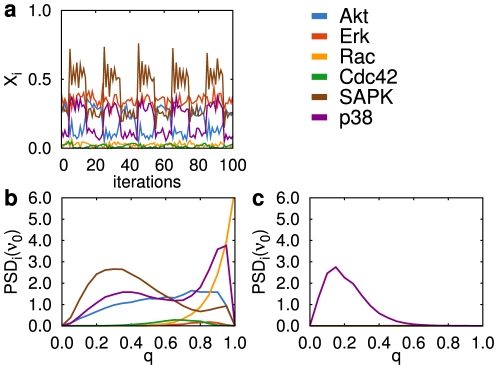
Network response to periodic stimulation of input node stress in the presence of chatter. (a) Temporal evolution of the population average of the output nodes (R = 201 cells) for a chatter level of 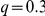 in the original network. Some species (Akt, SAPK and p38) oscillate at the frequency of the input signal. Power spectral density (PSD) at the input frequency obtained from the population average of each output node versus chatter level, 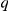, in the original network (b) and an AI randomized network (c).

Here we study the ability of the network to process and transmit *structured* information under different levels of background chatter. In order to do so, we examine the response of the network to the periodic stimulation of a specific input node, maintaining the rest of inputs at a constant chatter level (see examples of realizations and population activity for this type of input in Figure 1c). Contrary to what is observed for unstructured inputs (Figure 1b), the output signals obtained in these settings do have temporal structure. This is illustrated in Figure 3a, which shows the population average, 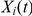, for all the output nodes upon periodic stimulation of the input node stress. Some outputs (Erk and Cdc42) do not show a significant response to the periodic modulation of stress, while others (Akt, Rac, SAPK, and p38) do oscillate at the period of this input.

In order to quantify the amount of periodicity in the network's response, we calculate the power spectral density (PSD) of each output node (see Figure S6). Figure 3b shows the value of this quantity at the frequency of the stress modulation, 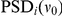, as a function of increasing 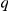 levels. This plot shows that the response at the input frequency changes in a nontrivial manner as a function of the chatter level (specially for Akt, Rac, SAPK, and p38). The different outputs reproduce the input periodicity in distinct ways, in some cases displaying their maximum response at intermediate 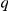 values. The stress-activated protein kinase (SAPK), for instance, seems to respond better to periodic stimulation by input stress at chatter levels close to 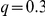. Another stress-activated protein, p38, is also an interesting example that presents two ranges of high response for chatters around 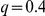 and 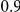. This behavior implies that the most responsive output to stress varies as the chatter level changes. In the particular example shown in Figure 3b, the most responsive output under increasing chatter values follows the sequence SAPK, Akt, p38 and Rac. In this sense, the network acts as a system capable of selecting its *dominant output* depending on the degree of background activity.

We have studied in detail the periodic stimulation of every input node of the fibroblast network, and have found that they all show the same qualitative phenomenology (see Figure S7), with the exception of the 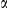 ligands. These ligands differ from the rest of inputs in that they represent generic pathways. For example, 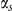 ligands correspond to the signals that use the 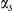 subunit of the G protein (which include epinephrin, glucagon, TSH, and more), while 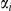 ligands (like acetylcholine, serotonin and angiotensin) use the 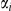 subunit, etc. They possibly fail to respond because their logic has somehow been altered during the generalization process. For clarity of presentation, we continue focusing hereafter on the periodic perturbation of stress only.

We now examine the extent to which the chatter-dependent ability of the network to select its response depends on its connectivity. To address this question, we now perform the same numerical experiment for the two randomizations of the network described previously (and shown in Figure S2). As in the case of unstructured inputs discussed in the previous section, AL networks in which the logic at all nodes is randomized (right column in Figure S2) are again insensitive to chatter, and in fact they do not respond to the periodic input at all (Figures S7 and S8). In contrast to the case of unstructured inputs, where AI networks did respond to the levels of chatter (Figure S5), we observe here that this second family of randomized networks are barely able to sense the periodic input, and are thus unable to show a sensitivity of the output to background chatter. A particular example of the response of such weakly randomized network to a periodic modulation of the stress input is shown in Figure 3c. Only p38 responds at all in this case. Other realizations of the randomization and the responses to other inputs are displayed in Figure S7, showing a similar behavior. This suggests that the topological structure of the network and the distribution of logic rules of the nodes are not sufficient for a successful information processing, but the original –specific– logic rules for each node are needed.

### Chatter level determines the information path

**Figure 4:**
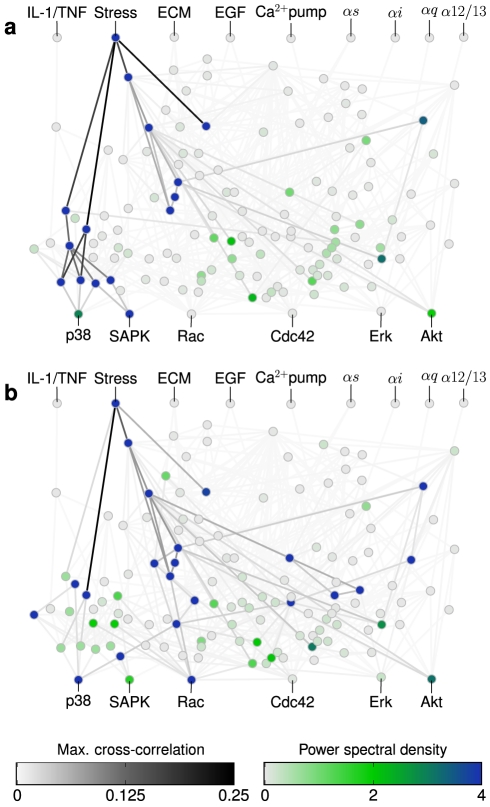
Chatter level determines the set of nodes that respond to the input modulation. Power spectral density at the input frequency (color coding of the circles) and maximum cross-correlation (in absolute value) between pairs of nodes (grey coding of the edges) for a periodic modulation of the stress input and two values of the background chatter level affecting the rest of inputs: (a) 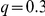, (b) 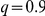.

Next we study how the information is transmitted from the stimulated input to the dominant output, and which nodes participate in this transmission. To that end, we calculate the power spectral density at the stimulation period for all network nodes, and the maximum cross-correlation (in absolute value) between the average signals of all pairs of nodes (see Supporting Text S1 for additional details). Figure 4 shows this information in the case of a periodic modulation of the input stress, and for two different values of the chatter level, 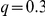 and 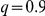, which correspond to the conditions for which SAPK and p38, respectively, are dominant outputs (Figure 3b). A common feature of both panels in Figure 4 is that there are several internal nodes that reproduce quite well the periodic input signal (i.e. they have high values of the power spectral density at the input frequency), and which are usually connected to each other by high cross-correlation values. However, there are also important differences between the two chatter levels. For instance, when 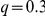 (Figure 4a), most of the nodes that transmit the signal from the stimulated node to the dominant output node (in this case, SAPK) are not so active for 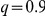 (Figure 4b), and a different set of nodes transmit the information from the stress input to p38, Rac and Akt, which now become dominant outputs. Together, these results show that chatter is able to select which output responds dominantly to a given input by determining the set of internal nodes that are most affected by the input. These nodes in turn signal downstream until a given output node is reached.

**Figure 5:**
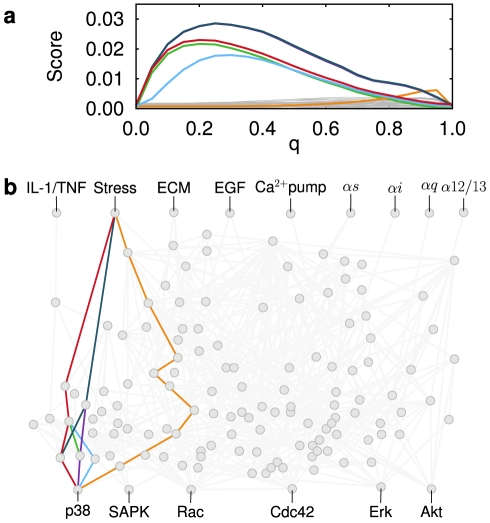
Paths of information flow for varying levels of chatter. (a) Information transmission scores (see text) of the dominant paths versus chatter level for a periodic modulation of the stress input, when considering p38 as output. (b) Edges defining these dominant paths.

Figure 4 shows that the chatter level sets which groups of internal interconnected nodes convey information from the inputs to the outputs. These groups of nodes and the links between them constitute preferred paths of information transmission. In order to characterize which of these paths are dominant in transmitting information, we resort to optimization algorithms of graph theory. For each stimulated input and chatter level, we assign a weight to each edge 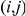 of the network equal to the inverse of maximum cross-correlation 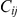 (see Supporting Text S1 for additional details). Then, for each of the network outputs we use a shortest path algorithm [31] to identify those paths going from the stimulated input to the considered output with the minimal sum of weights. This approach is well suited for our problem, as it penalizes large paths, and paths where at least one edge has a low cross-correlation. Each of the paths found using this method is assigned a score equal to the inverse of the sum of weights. Thus, the higher the score of a path, the higher the correlations of its constituent interactions. Those paths with highest score in terms of sums of these weights are what we define as *dominant paths*. In Figure 5a, we show the score of the best paths found going from input stress to p38 as a function of the chatter level 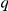 (see Figure S9 for the results corresponding to other input-output combinations). In Figure 5b, we show the nodes and interactions forming these paths. They are relatively short, as they usually involve between 3 and 7 intermediate species (structurally the network has 7 paths with 3 or less intermediate species from stress to p38, and 570 paths with 7 or less intermediate species). For low chatter levels, a group of paths emerges involving the MKK3 and MKK6 activation of p38. This group of paths is responsible for the first peak in the power spectral density of p38 shown in Figure 3c. At high levels of chatter, these paths fade out, and the oscillatory behavior of p38 (second peak in Figure 3c) becomes then due to inhibition by the MAP phosphatases (MKP), which in turn are activated through the adenyl cyclase (AC-cAMP) pathway. This is a specific prediction of our model, according to which the preferential pathway through which p38 is activated by stress changes with chatter. Since chatter can be varied by controlling the concentrations in the culture medium [32] of all input signals other than stress, it would be interesting to vary the medium composition of a fibroblast culture in a controlled way. The goal would be to measure the correlation between p38 activity and that of the main players of the two alternative pathways, e.g. MKK3 and AC, to check whether this correlation changes with medium composition. Similar predictions can be extracted for other input-output pairs.

### The network presents a balance between robustness and responsiveness

From the results of the previous section it is clear that different dominant paths emerge as a consequence of varying chatter levels. The remaining question is what happens to those internal nodes of the network not involved in the aforementioned paths when chatter level varies. To address whether they significantly change their processing capacity, we analyze the sensitivity to chatter variation of the power spectral density at the input frequency, 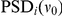, and of the maximum cross-correlation (in absolute value) between edges, 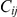. We call these two magnitudes, respectively, node sensitivity [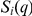 for node 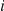] and edge sensitivity [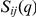 for the interaction pair 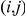] (see Supporting Text S1 for additional details).

**Figure 6:**
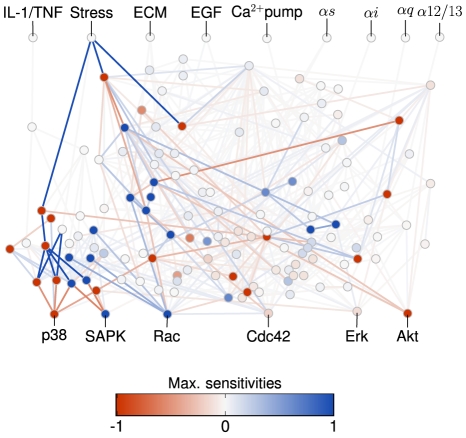
Maximum sensitivity of nodes and edges to chatter variation for periodic input stress. Red (blue) color scales for negative (positive) values of the sensitivity. Only a few nodes and edges present a high maximum (in absolute value) sensitivity. Most of these few nodes and edges are part of at least one of the paths previously identified as dominant (see Figures 4 and 5). The major part of nodes and edges not involved in information paths are robust to chatter, since they are mostly insensitive to it.

Figure 6 summarizes the results obtained in the case when the network is driven by an oscillating stress signal (see Figure S10 for other inputs). Both nodes and edges are colored according to the maximum (in absolute value) sensitivity for all chatter levels. Blue (red) color indicates a positive (negative) variation in the direction of increasing chatter. Color intensity indicates the magnitude of this maximum variation. In this figure, it can be observed that just a few nodes and a few of the 542 edges of the network have a significant variation of power and correlation when varying chatter levels. Note that most of the sensitive nodes and edges are involved in one or more dominant paths at a given chatter level (see Figures 4 and 5). Thus, while species belonging to paths involved in information transmission are sensitive to variations in the chatter level, the rest of the network nodes seem to be robust against these variations.

## Discussion

Cells live in environments whose composition affects the way in which they function. An example is the interstitial fluid (IF) surrounding cells in higher organisms, which affects processes as important as embryogenesis, tissue morphogenesis, remodeling and cancer progression [33]. The composition of the IF changes over time as a function of tissue irrigation rate, inflammation, and organ motility, for example. Modifications of the IF are known to affect fibroblasts [34], supporting the view that these cells are exposed to varying environments. While being subject to purely external sources of variation, cells also contribute to modifying their surroundings by secreting multiple signaling molecules themselves [35]. In a given physiological situation only a small subset of those signals will carry information relevant to the cell [36].

Noteworthily, the information-carrying signals are frequently dynamical, since oscillations in cell physiology are ubiquitous [37], and in many cases clearly periodic, driven for instance by regular biological rhythms such as those generated by circadian clocks [29], [38]. The remaining signals may constitute a source of background activity, or chatter, that is bound to affect the cellÕs response to the relevant inputs. Experimental evidence hints at the existence of signaling fluctuations in different cell types. Transient fluctuations in phosphate signaling, for instance, exist in yeast [39]. T cells, on the other hand, are known to be activatable by small numbers of T-cell-receptor (TCR) ligands [40], [41], and can therefore be expected to undergo strong fluctuations in TCR signaling [32]. In the particular case of fibroblasts, considered in this paper, fluctuations in extracellular pH are known to exist [42], and transient deactivation of ERK signaling (a pathway specifically considered in the model above) has been associated with cell cycle control [43]. The level of chatter will depend on many variables, including cell type, tissue, developmental stage, health status, etc. In this paper we numerically examine how the information transmission capabilities of a periodically stimulated human cell depend on the amount of background chatter.

At the experimental level, context-dependent signaling is beginning to be unraveled. For example, the Alliance for Cell Signaling (AfCS) recently compared the effect of 22 individually applied inputs (cytokines, GPCR ligands, TLR ligands, and tyrosine-kinase receptor ligands) upon 42 cell outputs (cytokine production, protein phosphorylation, calcium, and cAMP levels), to the effect of all possible pairwise combinations of those inputs [11]. According to the results of that study, only a few ligands are able to control cellular outputs independently from the other inputs. In contrast, most inputs act as modulators of signal transduction, providing the cell with the ability to perform *context-dependent signaling*. Our results fit well with these findings, as we see that the level of background activity of the input nodes determines the capability of the cell to respond to other inputs (in particular, to follow both unstructured and structured signals). In their work, Natajaran *et al.* [11] coin the term *interaction agent* to refer to the network circuits that couple different signaling pathways. They claim that such circuits would be silent in single ligand experiments and become active upon multiple input signaling, causing the non-additive effects observed for certain pairs of inputs. In our theoretical study we effectively observe different areas of the network being used at specific chatter levels, thus supporting the existence of these circuits.

Our results show specifically that a detailed signaling network, carefully compiled from published experimental data [15], responds in a nontrivial manner to background chatter, both intrinsically and in the presence of a periodic modulation of one of the inputs. This work extends the findings of Helikar and colleagues, who created the network in the first place and studied its stationary response to different input levels for increasing intensities of noise [15]. They concluded the network divides biological stimuli into categories, since it reduces the full range of possible external inputs to a limited number of cell responses, in a manner that is robust to noise. We divert from the work of Helikar *et al.* in that we focus on the dynamics of the system. Having recently explored the relaxation time and frequency response of the network [26], we now show that chatter is able to enhance the response of certain outputs to a given input when tuned to optimal levels. Given that chatter controls the amount of stochasticity acting upon the network, this is a situation reminiscent of stochastic resonance, a phenomenon in many physical and neurosensory systems by which the detection of a weak signal is enhanced by noise [44]. Our simulations have been performed using synchronous updating but, as shown in Supporting Text S1 and in Figure S11, our results are qualitatively unaltered when the updating is asynchronous (whose main effect is the destruction of deterministic attractors, which is also caused by chatter). We also note that the temporal character of the chatter is relevant for the phenomena reported here, as discussed in Supporting Text S1 and in Figure S12.

Recent studies have shown that signaling networks prioritize dynamic range over signal strength [45]. This entails a linear relationship between the input signal and the output response of the network, which ensures that the reaction of the network to an oscillatory input will also be oscillatory with the same main frequency, for a large wide range of input amplitudes. Our results fit well with this finding, and extend it by assigning a relevant role to the background chatter coming from other input nodes, which enhances the frequency response. It would be interesting to extend these studies to the situation in which more than one information-carrying signal act upon the system, following the recent experimental studies of Natajaran *et al.* [11] and Hsueh *et al.* [36] discussed above, which have revealed synergistic effects in signal integration.

Our results also show that varying chatter levels allow the network to select which output nodes respond preferentially to a given input. Indeed, output switching is achieved via a mechanism that places few requirements on the temporal structure of contextual, non-specific signals. Thus, we conjecture that cells could use environmental noise (to which they are unavoidably subject) to choose among alternative information routes, and eventually among different cellular responses. Randomized versions of the original network in which the topology of the connections is maintained -and only the integration rules at the nodes are altered- fail to reproduce this property, indicating that the chatter-driven selectivity reported here is fine-tuned to the specific architecture and logic of the experimentally-supported network.

Concurrent with the ability of background chatter to select the dominant output for a given input, chatter also selects the network path through which information is transmitted. The nodes belonging to these preferred paths can be expected to form the *classifier hyperspace* proposed by Oda and Kitano in their study of the Toll-like receptor signaling pathway [5]. These preferred paths are sensitive to chatter and allow transitions between different information processing scenarios that underlie different output responses. In that way, a given signaling network can have multiple working states that are selected by the background chatter. The rest of the network nodes not belonging to the preferred paths, on the other hand, remains insensitive to chatter. In that way, we can conjecture that signaling networks have a built-in balance between responsiveness and robustness within their coupling architecture, and this balance is modulated by background chatter. Taken together, the results presented here indicate that background activity levels are key for determining the response of the cell to a given input, by allowing the emergence of novel system-level properties such as information routing, output switching, and context-dependent signaling.

## Supporting Information

Figure S1 **Annotated fibroblast network.** Specific information about the update rule for each node can be found at http://mathbio.unomaha.edu/Database.
(EPS)

Figure S2 **Network randomization.** Two methods of randomizing signal integration at the network nodes. The update rule of one of the nodes of the original network is shown as an example (left column). The first randomization method (*altered-logic*, middle column) consists on shuffling the output of the update rule, in such a way that the logic of the logic gate changes but the number of active output states is maintained. The second randomization method (*altered-input*, right column) consists instead on shuffling the inputs of the network node while maintaining the logic of the update rule (the logic is given below the truth table in each case).
(EPS)

Figure S3 **Topological comparison of the networks.** Distribution of the number of canalizing functions for the AL networks, as defined in Figure 7. The arrow marks the number of canalizing functions of both the original and the AI networks.
(EPS)

Figure S4 **Response of the biological and randomized networks to unstructured inputs.** Temporal average of the output population activities for increasing chatter level for unstructured inputs. (a) Original network; (b–f) five out of 100 realizations of the altered-logic network models, in which the logic rules at the nodes are changed randomly (see Figure 7); (g–k) five realizations out of 100 of the altered-input network models, in which the logic rules are maintained but the inputs are shuffled randomly.
(EPS)

Figure S5
**Quantifying the responsiveness of randomized networks to unstructured inputs.** Distribution of the number of output nodes that respond to the unstructured inputs of Figure 9, for 100 realizations of each of the two types of network randomizations described in Figure 7. A node is considered responsive when the dynamic range of the average activity 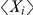 (difference between maximum and minimum values for varying 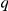, as in Figure 9) surpasses a given threshold, taken here equal to 0.25 in the units of the y-axis of Figure 9, without loss of generality. The arrows indicate the number of responsive inputs of the original biological network.(EPS)

Figure S6
**Network response to periodic stimulation of the input node stress in the presence of chatter.** Power spectral density of the average activity shown in Figure 3a of the main text. The first frequency peak corresponds to the frequency of the input signal (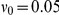). These power spectral densities are calculated for series of 1600 iterations, after dismissing a transitory period of 160 time iterations in the data.(EPS)

Figure S7
**Response of the biological and randomized networks to structured inputs.** Power spectral density at the stimulation frequency of the population activities of the output nodes (see legend in Figure 9) for five structured inputs (calcium pump, 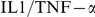, extracellular matrix, EGF and oxidative stress), as a function of chatter level, 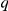. (a) Wild type network; (b–f) five altered-input networks; (g–k) five altered-logic networks.(EPS)

Figure S8
**Quantifying the responsiveness of randomized networks to structured inputs.** Distribution of the number of output nodes that respond to a modulation of the stress input (Figure 12), for 100 realizations of each of the two types of network randomizations described in Figure 7. A node is considered responsive when the dynamic range of the power spectral density at the stimulation frequency (difference between maximum and minimum values for varying 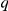, as in Figure 12) surpasses a given threshold, taken here equal to 3% of the maximum PSD of the input, without loss of generality. The arrows indicate the number of responsive nodes of the original biological network.(EPS)

Figure S9 **Paths of information flow between different input-output pairs, and varying levels of chatter.** Paths with highest correlation for all chatter levels (top panels in each figure set) and correlation scores of those paths as chatter increases (bottom panels in each figure set). Figure sets (a) to (e) correspond to the five different specific inputs, and within each set the columns correspond to the different outputs.
(EPS)

Figure S10 **Network sensitivity to chatter.** Maximum sensitivity of nodes and edges when no structured input is present (a) and for five structured inputs (b–f, input listed right before each plot). Red (blue) color scales for negative (positive) values of the sensitivity. Only a few nodes and edges present a high maximum sensitivity in absolute value. Most of these few nodes and edges are part of at least one of the paths previously identified as dominant. The major part of nodes and edges not involved in transmission information paths are robust to chatter as they do not have large sensitivities to it.
(EPS)

Figure S11
**Robustness to asynchronous updating.** Network response to periodic stimulation of input node stress (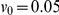) in the presence of chatter, quantified by the power spectral density of the output nodes (see legend in Figure 9), for three different asynchronous updating methods (see text). The power spectral densities are calculated for time series of 1600 iterations, after dismissing a transitory period of 160 iterations in the data, and for a population of size R = 201 for each 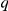. (a,b) Results for the two-step partial randomization update scheme, with a probability of being updated in the first step equal to 0.5 (a) and 0.05 (b). (c) Results for the complete randomization update scheme.(EPS)

Figure S12
**Role of temporal variation of the chatter.** Fraction of responsive cells (realizations) versus chatter level 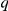 for (a) 201 realizations of temporally varying chatter, as considered in the main text, and (b) 2001 realizations of quenched chatter (see Supporting Text S1 for a definition of quenched chatter). A cell is considered responsive if the maximum value of the cross-correlation function between input (stress) and output (color coded according to the legend in Figure 9) surpasses 6.25% of the perfect score.(EPS)

Text S1 **Supporting Text.**
(PDF)
