# Supplementary material for: Information Routing Driven by Background Chatter in a Signaling Network
Source: PLoS Comput Biol. 2011 Dec 8;7(12):e1002297. doi: 10.1371/journal.pcbi.1002297 (PMC3234210; doi:10.1371/journal.pcbi.1002297)
Supplement: Text S1 — Supporting Text. (PDF) [file pcbi.1002297.s013.pdf]

SUPPORTING TEXT S1

Information routing driven by background chatter in a signaling network

Núria Domedel-Puig[1,*], Pau Rué[1,*], Antonio J. Pons[1], Jordi García-Ojalvo[1,¶]
1 Departament de Física i Enginyeria Nuclear, Universitat Politècnica de Catalunya, Terrassa, Barcelona, Spain
∗ Equal contribution
¶ E-mail: jordi.g.ojalvo@upc.edu

S1 Boolean network modeling

Boolean networks (BN), introduced by Kauffman in 1969, are defined as sets of dynamical units, called nodes, which are connected by interaction edges. The dynamic evolution of a Boolean network is characterized by logic operations which act upon binary state variables defined in each node. Thus, the state of every node i at time t, $x_i(t)$, is either active ($x_i(t) = 1$) or inactive ($x_i(t) = 0$) and all nodes are updated synchronously according to a set of node-specific logic rules [S1]. Here we will work with non-autonomous BNs, in which two types of nodes exist according to its dynamics: *internal* nodes and *input* nodes. In particular, every internal node i has an associated logic rule, f_i, which determines the new state $x_i(t)$ at time t from the states of its k_i incident nodes at time $t-1$:

$$x_i(t) = f_i(x_i^1(t-1), \ldots, x_i^{k_i}(t-1)) \qquad \text{(S1)}$$

The state of the *input* nodes, in contrast, does not depend on the state of other nodes but is given externally. Here we set these states using periodic and random value sequences. The update rules, $f_i, i = 1, \ldots, N$, can be specified either as a truth table or as a composition of basic logic functions (ANDs, ORs, NOTs ...). Simulations obtained using these functions are, in general, more efficient than those obtained using truth tables. To speed up the simulation runtime, we transformed the original truth tables [S2] to the corresponding equivalent logic functions using an adapted version of the Quine-McCluskey algorithm [S3]. We implemented the Boolean network simulator in Python (the custom-made code is available at http://code.google.com/p/bnsim).

In order to generate the stochastic input sequences, we use Bernoulli distributions with success probability equal to q, which we define as the *chatter level*. Bernoulli distribution sequences are obtained using a standard Mersenne twister pseudo-random number generator [S4].

S2 Network properties

The BN model used in this work was obtained from the MathBio web in the form of a list of nodes with an associated truth table (http://mathbio.unomaha.edu/Database). This network describes the signaling pathways present in a prototypical human fibroblast, and was built by Helikar *et al.* [S2] using the information obtained from a large body of literature and is formed by 9 input nodes that feed 130 internal nodes. Input nodes represent signals of varying nature, namely stress signals (oxidative stress and the IL1/TNF$-\alpha$ route), growth factors (EGF), calcium channels (Ca^{2+} pump), signaling by extracellular matrix components (ECM), and by ligands that use G-protein coupled receptors (one for each subtype of G protein α subunit: α_s, α_i, α_q, $\alpha_{12,13}$). The average number of inputs per node (in-degree) and the average number of nodes affected by one given node (out-degree) are both 3.9. The structure of this network is very different from classical Kauffman networks [S1]. Specifically, neither the in-degree nor

the out-degree distributions are uniform throughout the network, contrary to Kauffman networks. For instance, while there are 21 nodes with a single input, the Src protein is affected by 11 different nodes (its in-degree is equal to 11) and affects 28 downstream nodes (its out-degree is equal to 28). In fact, Src is the protein with highest out-degree and closeness centrality. Other interesting nodes are Rac, which integrates information from 13 species (maximum in-degree) and Erk, which is the node that participates the most in the shortest paths among all pairs of species (highest betweenness centrality). Another important characteristic of this network is the high proportion of *canalizing functions* [S2, S5]. A node rule f_i is said to be canalizing for a given input if its output is completely determined by at least one specific value of that input, independently of the values of the remaining inputs. As an example, consider the canalizing rule $f(x_1, x_2, x_3) = x_1 \,\mathrm{OR}\, (x_2 \,\mathrm{AND}\, x_3)$. Note that, with this logic, whenever x_1 takes the value 1, the output is 1, independently of the truth values of x_2 and x_3. In this case, f is *canalizing* for x_1, and x_1 is a *canalizing input* of f. A total of 96 nodes in the fibroblast network are canalizing for at least one of their inputs. In addition, 168 from the 542 interactions are canalizing. For instance, Src protein acts in a canalizing manner for 8 of its immediately downstream proteins while PI4K, Mekk1 and PI3K have 4 canalizing inputs. There are no paths from inputs to specific outputs built fully from canalizing functions, except the direct link going from ECM to Rac.

S3 Simulation conditions

For each simulation condition considered in our study, we have run $R = 201$ different realizations of the dynamics. Each of these realizations can be regarded as the time evolution of a particular cell, and differs from the others in the initial state of all the internal nodes, which are set randomly, and by the different realizations of input chatter. The population activity is obtained as the average over individual realizations, $X_i(t) = \frac{1}{R}\sum_{\mathrm{cell}=1}^{R} x_{i,\mathrm{cell}}(t)$. This quantity corresponds to the probability of the node i being active at time t in the limit of large R.

In this work we perform two types of numerical experiments. In the first type, we set all 9 inputs to a given (constant) chatter level and observe the population activity of the output nodes. In the second type, we force one input to be a periodic sequence of ones and zeros (representing an alternative switch ON and OFF of its activity) and set the rest of inputs to a fixed chatter level. We refer to this periodically switching input as the stimulated input or *structured signal*. The period chosen, $T_0 = 20$ (ten ones followed by ten zeros), is of the same magnitude as the average transient time needed to reach periodic attractors for fixed inputs [S6].

All simulations in this work have been run for 1600 iteration steps. The first 160 iteration steps were disregarded in order to ensure that no transient dynamics were present in the subsequent analysis.

S4 Network randomization

We have taken two distinct approaches to randomize the original network (see Figure S1), keeping in both cases the topology of the network constant, while varying the rules of the nodes. In the first case (*altered-logic* networks, AL), the network is randomized by sampling a completely new random rule for each node, with the only constraint that the number of ones in the truth table is the same as in the original table. The algorithm used to generate this kind of network is the following: for every internal node, i, of the network, we take its original truth table, consisting of 2^{k_i} entries, and count the number of entries that lead to an active state of node i, say $n(i)$. For the new network, we reassign the $n(i)$ entries that lead to the active state for this node. Taking into account that there are $\binom{2^{k_i}}{n(i)}$ different possible logic rules for every node i, we see that the number of possible networks produced in this way is extremely large. These networks are structurally equivalent to the original BN in the sense that the links forming them are maintained. However, the distribution of logic rules is completely different. In particular,

the distribution of canalizing functions for this family of networks is very narrow and is centered on 63 canalizing functions, while the original network has exactly 96 (see Figure S3).

We built a second type of randomized network by shuffling the inputs of the logic rules of each internal node (*altered-input* networks, AI). In this case, the procedure for generating the network is the following: for each internal node, i, with k_i inputs, we randomly draw a permutation, $\sigma : \{1, \ldots, k_i\} \to \{1, \ldots, k_i\}$, defining the new logic rule, $\bar{f}_i$, as:

$$\bar{f}_i(y_1, \ldots, y_{k_i}) = f_i\left(y_{\sigma(1)}, \ldots, y_{\sigma(k_i)}\right) \tag{S2}$$

Even though the roles of the input species for every node are changed, this building procedure ensures that the type of logic rule for every node is the same as in the original network. The number of canalizing functions of the network is preserved. Thus, the type of networks produced in this way, while having different connectivity than the original network, are topologically very similar to it. Again, taking into account that there are k_i! different logic rules for every node, we see that the number of possible networks produced following this algorithm is very large. We have simulated and analyzed 100 random networks of each type.

S5 Robustness to asynchronous updating

As explained in the main text, our simulations have been performed using synchronous updating. Asynchronous updating has been shown to destroy some of the attractors found in networks previously simulated with a synchronous-updating framework (see, for instance, Refs. [S7, S8]). We have shown in a previous publication [S6] that when input nodes are subject to variability, attractors cannot be reached because states are forced to change exogenously (except for $q = 0$ and $q = 1$). Therefore, the effect of an asynchronous updating scheme upon the network attractors is similar to the effect of chatter. We can thus expect the behavior reported here not to be qualitatively altered when the updating is asynchronous. To verify this point, we have implemented different asynchronous updating schemes in our model.

In a first asynchronous method, the updating order of the nodes is randomized at each iteration, and all nodes of the network are updated sequentially following this order. In that way, one iteration in this scheme corresponds to updating once the whole network, as in the synchronous scheme. However, this is an extreme change with respect to the synchronous method, in the sense that we switch from a parallel updating order (synchronous update) to the opposite scenario in which the asynchronous updating sequence is specific to each iteration. To examine an intermediate situation, we have implemented a second asynchronous update method in which every iteration is composed of two steps. During each of these steps, a different partition of nodes is updated synchronously. Nodes are assigned to the early-update partition according to a given probability p, which we set to 0.95 to model a soft asynchronous updating (close to the purely synchronous case defined by $p = 1$), while $p = 0.5$ models a more asynchronous method.

Both asynchronous update schemes described here share the fact that they select the nodes randomly at each iteration. This fact strongly suppresses the ability for the structured ordering coming from the periodic input to systematically percolate spuriously through the network. However, the results (see Figure S11) show that the nontrivial influence of chatter on the response to a periodic input persists in the presence of asynchronous updating. The transmission of the input signal in this case can thus be attributed only to the robustness of the dynamical behavior with respect to these causal destructive updates.

S6 Role of temporal variation

As discussed in the preceding section, the temporal variation associated with chatter prevents the system from becoming trapped in attractors, in which some nodes might become permanently unresponsive to

the input signal. Thus the response of the network to chatter goes beyond a mere sensitivity to different average levels of network activity in different nodes. To verify this, we run simulations in which the chatter is fixed to its initial value (quenched chatter), and calculate the number of cells (i.e. realizations) that are responsive to the input, comparing it with the case of temporally variable chatter (the one considered in the main text). A cell is considered responsive if the maximum value of the cross-correlation function between the input signal and a certain output response is larger than a given threshold (chosen here equal to 6.25% of the maximum correlation, without loss of generality). Our results, displayed in Figure S12, show that the fraction of responsive cells for a given input is substantially smaller in the case of quenched chatter, for all chatter levels. Thus temporal variability of the chatter plays a relevant role in the behavior reported.

S7 Methods

S7.1 Power spectral density and maximum cross-correlations

When one of the inputs carries a structured signal, we are interested in identifying those nodes of the network that are oscillating at the frequency of that input. To characterize that response we estimated the power spectral density (PSD) of the time series of the population average activities of all nodes, using the Welch periodogram method. The network nodes that present a clear peak centered at the stimulation frequency in the power spectrum are considered to be responsive to that input. We quantify this responsiveness in terms of the value of the PSD at the input frequency, $\nu_0 = 1/20$.

Besides identifying the nodes that reproduce the structured signal, we want to estimate which pairs of interacting nodes, i and j, have correlated signals. To quantify this, we measure the cross-correlation of the average activity,

$$c_{ij}(\tau) = \frac{1}{T-\tau} \sum_{t=\tau}^{T} \big(X_i(t) - \langle X_i \rangle\big)\big(X_j(t-\tau) - \langle X_j \rangle\big) \quad \text{(S3)}$$

where $\langle \cdot \rangle$ represents the standard time average. The cross-correlation function $c_{ij}(\tau)$ measures how similar the population signals X_i and X_j are when the second signal presents a temporal delay of τ with respect to the first one. Although it is known that correlation does not imply causality, if two linked nodes are correlated, it is often reasonable to assume that the source node transmits information to the target node of the link. For each edge of the network, a simple measure of information transmission is, hence, the maximum value of the cross-correlation with respect to the delay τ, $C_{ij}(q) = \max_{\tau \in \{0,\ldots,T_0\}} |c_{ij}(\tau)|$ where T_0 is the period length and the absolute value in the formula accounts for phase inversions.

S7.2 Identification of dominant paths

The chatter level establishes groups of interconnected internal nodes, which define paths that convey information from input to outputs nodes (see main text). In order to characterize which of these paths are dominant in transmitting information, we resorted to optimization algorithms of graph theory. To apply these algorithms, we had to assign a weight to each edge of the network. A simple measure of information transmission through each edge is the maximum value of the cross-correlation, which can thus be used as a weight:

$$C_{ij}(q) = \max_{\tau \in \{0,\ldots,T_0\}} |c_{ij}(\tau)| \quad \text{(S4)}$$

We built a directed weighted graph using a measure based on this assignment. The idea is to determine the paths going from each input node to each output node in such a way that each edge forming this path has a high enough maximum cross-correlation. Hence, every path going from a node i to a node j is formed by a set of edges, $\{(i_1, i_2), (i_2, i_3), \ldots, (i_{L-1}, i_L)\}$, in such a way that $i_1 = i$ and $i_L = j$. The

criterion we chose was to assign a weight equal to the inverse of C_{ij}, $W_{ij} = C_{ij}^{-1}$, to each edge (i, j), and to look for paths that minimize the sum of these weights. We define a *correlation score* or cost function, $\mathcal{S}$, for each path, as the inverse of the sum of weights along it:

$$\mathcal{S}\left(\{(i_1, i_2), (i_2, i_3), \ldots, (i_{L-1}, i_L)\}\right) = \left(\sum_{k=1}^{L-1} W_{i_k, i_k+1}\right)^{-1} \tag{S5}$$

To obtain the paths with maximum correlation score, we used the $K-$th shortest path algorithm [S9], which identifies the first K paths with minimum sum of weights, and thus the maximum path score ($K-$th shortest path algorithm with $K = 10$ shortest paths). This approach is well suited for our problem because it penalizes large paths, in which case more terms are added to the path score, and paths where at least one of the edges has a low maximum cross-correlation value, $C_{i_k, i_{k+1}}$. We set the number of top shortest paths to be found, K, to a high enough value, such as $K = 10$, which ensures that all paths with high scores are identified. After finding the highest score paths for all q values, a threshold-based filtering is set to find those paths with scores higher than a certain level, $\mathcal{S}_c$.

S7.3 Definition of node and edge sensitivities

As in many dynamic systems, a measure of how sensitive the response of the system is to a certain parameter can be introduced for our model. In particular, we can define measures of sensitivity to chatter level for both nodes and edges of the network. We define the sensitivity of nodes to chatter as the variations of power spectral density at the input frequency with respect to variations in the chatter. In mathematical terms, the sensitivity of node i can be defined as $S_i(q) = \frac{\partial}{\partial q}\mathrm{PSD}_i(\nu_0)|_q$. Likewise, we define the sensitivity of edges to chatter variations as $S_{ij}(q) = \frac{\partial}{\partial q} C_{ij}(q)|_q$. Both derivatives are estimated by centered differences with a differentiation step of $\delta q = 0.05$.

Supporting References

S1. Kauffman S (1969) Metabolic stability and epigenesis in randomly constructed genetic nets. *J Theor Biol* 22:437–467.

S2. Helikar T, Konvalina J, Heidel J, Rogers J (2008) Emergent decision-making in biological signal transduction networks. *Proc Natl Acad Sci USA* 105:1913–1918.

S3. Quine WV (1952) The problem of simplifying truth functions. *Am Math Mon* 59:521–531.

S4. Matsumoto M, Nishimura T (1998) Mersenne twister: a 623-dimensionally equidistributed uniform pseudo-random number generator. *ACM T Model Comput S* 8:3–30.

S5. Kauffman S, Peterson C, Samuelsson B, Troein C (2004) Genetic networks with canalyzing Boolean rules are always stable. *Proc Natl Acad Sci USA* 101:17102–17107.

S6. Rue P, Pons AJ, Domedel-Puig N, Garcia-Ojalvo J (2010) Relaxation dynamics and frequency response of a noisy cell signaling network. *Chaos* 20:045110.

S7. Klemm K, Bornholdt S (2005) Stable and unstable attractors in Boolean networks. *Phys Rev E* 72:055101(R).

S8. Greil F, Drossel B (2005) Dynamics of critical Kauffman networks under asynchronous stochastic update. *Phys Rev Lett* 95:048701.

S9. Martins EQV, Pascoal MMB (2003) A new implementation of Yen's ranking loopless paths algorithm. *4OR - Q J Oper Res* 1:121–133.
